# Biofilm Formation and Adherence Characteristics of *Listeria ivanovii* Strains Isolated from Ready-to-Eat Foods in Alice, South Africa

**DOI:** 10.1100/2012/873909

**Published:** 2012-12-26

**Authors:** Mirriam E. Nyenje, Ezekiel Green, Roland N. Ndip

**Affiliations:** ^1^Department of Biochemistry and Microbiology, Faculty of Science and Agriculture, University of Fort Hare, PMB X1314, Alice 5700, South Africa; ^2^Department of Microbiology and Parasitology, Faculty of Science, University of Buea, P.O. Box 63, Buea, Cameroon

## Abstract

The present study was carried out to investigate the potential of *Listeria ivanovii* isolates to exist as biofilm structures. The ability of *Listeria ivanovii* isolates to adhere to a surface was determined using a microtiter plate adherence assay whereas the role of cell surface properties in biofilm formation was assessed using the coaggregation and autoaggregation assays. Seven reference bacterial strains were used for the coaggregation assay. The degree of coaggregation and autoaggregation was determined. The architecture of the biofilms was examined under SEM. A total of 44 (88%) strains adhered to the wells of the microtiter plate while 6 (12%) did not adhere. The coaggregation index ranged from 12 to 77% while the autoaggregation index varied from 11 to 55%. The partner strains of *S. aureus*, *S. pyogenes*, *P. shigelloides*, and *S. sonnei* displayed coaggregation indices of 75% each, while *S*. Typhimurium, *A. hydrophila*, and *P. aeruginosa* registered coaggregation indices of 67%, 58%, and 50%, respectively. The ability of *L. ivanovii* isolates to form single and multispecies biofilms at 25°C is of great concern to the food industry where these organisms may adhere to kitchen utensils and other environments leading to cross-contamination of food processed in these areas.

## 1. Introduction

In nature, bacterial cells are most frequently found in close association with surfaces and interfaces, in the form of multicellular aggregates embedded in an extracellular matrix generally referred to as biofilms [1]. Biofilms are usually heterogeneous; in that they contain more than one type of bacterial species, but they can be homogeneous in cases such as infections and medical implants [2]. Microbial biofilms pose a challenge in clinical and industrial setting especially in food processing environments where they act as a potential source of microbial contamination of foods that may lead to spoilage and transmission of foodborne pathogens [3, 4]. They can also compromise the cleanliness of food contact surfaces and environmental surfaces by spreading detached individual microorganisms into the surrounding environment [5].

Environmental conditions in food production areas including the presence of moisture, nutrients, and inocula of microorganisms from the raw materials might favour the formation of biofilm. Furthermore, when food processing equipments are not easily cleaned due to its design and food particles not completely removed, the particles aid in the formation of biofilms by providing a coat that not only provides the biofilm with nutrients but also a surface to which it can easily stick on [6]. Once biofilms have formed on food processing surfaces, they are hard to eliminate often resulting in persistence and endemic population.

Biofilms offer their member cells several benefits, including channeling nutrients to the cells and protecting them against harsh environments. In particular, it has been noted that cells within biofilms are more resistance to antibiotics, disinfectants, and to host immune system clearance than their planktonic counterparts [3, 7]. Several mechanisms account for this increased antibiotic resistance, including the physical barrier formed by exopolymeric substances, a proportion of dormant bacteria that are inert toward antibiotics, and resistance genes that are uniquely expressed in biofilms [8]. Outbreaks of pathogens associated with biofilms have been related to the presence of species of *Listeria*, *Yersinia*, *Campylobacter*, *Salmonella*, *Staphylococcus*, and *Escherichia coli* O157 : H7. These bacteria are of special significance in ready-to-eat and minimally processed food products, where microbiological control is not conducted in the terminal processing step [6].

*L. monocytogenes* and *L. ivanovii* are potential pathogens of listeriosis, a rare but serious disease with a high mortality rate of 30% in pregnant women or immunocompromised individuals [9–11]. *Listeria *strains have been reported to survive for months to years in food processing environments and, thus, colonize various food products leading to food contamination [12]. *L. monocytogenes* biofilms in food processing plants have been widely studied [13]. However, there is a dearth of information on the ability of *L. ivanovii* to form biofilm; this might be due to the fact that it rarely causes human illnesses due to its low prevalence in the environment. Nonetheless, recent studies in the environment of the present study have reported high prevalence of the organism in wastewater effluents and various ready-to-eat foods [14, 15], suggesting that the organism might be endemic in the area. Therefore the present study was carried out to investigate the ability of* L. ivanovii *isolates to exist as biofilm structures, in an effort to establish the factors for this endemicity.

## 2. Materials and Methods

### 2.1. Biofilm Formation and Quantification

The biofilm forming ability test was done in accordance with the method of Stepanovic et al. [16]. *L. ivanovii *isolates obtained from different food sources as reported in our previous study [15] were cultured on Nutrient agar (Oxoid, Basingstoke, England) and plates were incubated at 37°C for 24 hours. Few single colonies were suspended in sterile saline to a turbidity standard comparable to a 0.5 McFarland. The suspension was vortexed for 1 minute from which 20 *μ*L was pipetted into a 96-well U-bottomed microtiter plate (Greiner Bio-one GmbH, Germany) containing 180 *μ*L of Brain Heart Infusion (BHI) broth (Oxoid, Basingstoke, England). The plates were incubated aerobically for 24 hours at 25°C ± 2°C. After incubation, the contents of the wells were decanted into a waste container and each well was washed three times with 200 *μ*L of sterile normal saline. Following every washing step, the well were emptied by carefully aspirating the content into a waste container and the plates were left to dry overnight in an inverted position before they were fixed with hot air at 65°C for 1 hour. Plates were stained with 150 *μ*L of 1% crystal violet for 30 minutes; the excess stain was aspirated and plates rinsed off by placing them under running tap water until the washings were free of the stains. The plates were left to dry at room temperature in an inverted position overnight before resolubilizing the dye bound to adherent cells with 150 *μ*L of 33% (v/v) glacial acetic acid; the optical density (OD) of each well was measured at 595 nm using a microtiter plate reader (SynergyMx, Biotek^R^, USA). Reference strains of *P. aeruginosa* ATCC 15442 and *S. aureus* NCTC 6571 were used as positive controls while negative control well contained broth only. Tests were performed in triplicates on three occasions, the results averaged, and biofilms quantified as nonadherent, weakly adherent, moderately adherent or strongly adherent. 

### 2.2. Autoaggregation and Coaggregation Assays

Twelve (three each nonadherent, weakly adherent, moderately adherent, and strongly adherent) *L. ivanovii* isolates and seven reference strains (*S. aureus* NCTC 6571, *S. pyogenes* A ATCC 49399, *S.* Typhimurium ATCC 13311, *P. aeruginosa* ATCC 15442, *P. shigelloides* ATCC 51903, *A. hydrophila* ATCC 35654, and *S. sonnei* ATCC 29930) were used for these assays. The bacteria strains were grown separately in 20 mL of BHI broth at 37°C for 48 hours. Cells were harvested by high-speed centrifugation (11,000 ×g for 10 min) and washed twice in 3 mM NaCl containing 0.5 mM CaCl_2_. Subsequently, the cells were resuspended in the same solution (3 mM NaCl containing 0.5 mM CaCl_2_) and centrifuged at 650 ×g for 2 min, and the supernatant carefully aspirated and discarded into a waste container. The OD of the cell suspension was measured and adjusted to 0.3 using an automated spectrophotometer (Optima Scientific V-1200) at a wavelength of 660 nm; the cell suspension was used for coaggregation assay. Equal volumes (1 mL each) of the coaggregating partners were mixed and the OD (OD_Tot_) of the mixture was immediately read at 660 nm before incubation at room temperature for 2 hours. Subsequently, the tubes were centrifuged at 2,000 rpm for 2 min and the OD of the supernatant (OD_s_) measured at the same wavelength (660 nm) [17].

The degree of coaggregation of the paired isolates was determined using the equation

(1) $$\begin{array}{c} \%\;\text{coaggregation}=\frac{{\text{OD}}_{\text{Tot}}-{\text{OD}}_{\text{s}}}{{\text{OD}}_{\text{Tot}}}\times 100. \end{array}$$

For autoaggregation assay, the individual bacterial suspension adjusted to an OD of 0.3 was incubated at room temperature for 1 hour and the cell suspension centrifuged at 2000 rpm for 2 minutes. The supernatant (2 mL) was transferred into a cuvette and the OD measured at 660 nm.

The degree of autoaggregation was calculated as follows:

(2) $$\begin{array}{c} \%\;\text{autoaggregation}=\frac{{\text{OD}}_{0}-{\text{OD}}_{60}}{{\text{OD}}_{0}}\times 100. \end{array}$$

OD_0_ refers to the initial OD of the organism, and OD_60_ is the OD of the supernatant after 60 min of incubation.

### 2.3. Characterization of Biofilm Formation Using Scanning Electron Microscope

The biofilms were further examined using scanning electron microscope (SEM) according to the method previously described by Greetje et al. [18] with some modifications. A representative of the biofilm forming strain population was studied. Briefly, a microscope cover slip (22 × 22 mm) on a glass slide was placed in a petri dish half filled with BHI broth. Subsequently, a few colonies of *L. ivanovii* were transferred into the BHI broth and incubated at 25°C for 72 hours. For the coaggregation assay, the partner isolate was added after 1 hour of incubation. The cover slips were washed three times with normal saline before fixing with 2.5% (w/v) glutaraldehyde solution for 1 hour. Subsequently, the samples were dehydrated in a series of 20, 40, 60, 80, and 99.5% ethanol solution for 30 min in each concentration. Finally the samples were postfixed in 1% Osmium tetroxide (OsO_4_), critical point-dried using CO_2_, and sputter-coated with Gold palladium using Elko 1B.3 ion coater before viewing with the SEM (Japan Electron Optical Laboratories JSM-6390LV).

#### 2.3.1. Data Analysis

Tests were done in triplicate on three separate occasions and the results averaged. The cutoff OD (OD_C_) for the microtiter plate test was defined as three standard deviations above the mean OD of the negative control. Isolates were classified as follows: OD ≤ OD_C_  = nonadherent, OD_C_ < OD ≤ (2 × OD_C_)  = weakly adherent; (2 × OD_C_) < OD ≤ (4 × OD_C_)  = moderately adherent, and  (4 × OD_C_) < OD  = strongly adherent [17].

## 3. Results

### 3.1. Microtiter Adherence Assay

The biofilm formation ability of 50 *L. ivanovii* strains is summarized in Table 1. Variations in biofilm formation were observed. A total of 44 (88%) strains adhered to the wells of the microtiter plate while 6 (12%) did not adhere. The majority of the isolates demonstrated weak (44%) and moderate (34%) adherence while only 5 (10%) strains strongly adhered to the wells. The optical density range of nonadherent and strong adherent isolates was 0.332–0.503 and 2.32–3.846, respectively.

### 3.2. Coaggregation and Autoaggregation

Coaggregation occurred to varying degrees between all the seven partner strains and *L*. *ivanovii* isolates. The coaggregation index ranged from 12 to 77% while autoaggregation ranged from 11 to 55%. Some strains which strongly adhere to the wells were equally able to stick to each other (autoaggregation of 32–55%); this was followed by moderate (35–41%) and weak adherent (30–46%) strains while nonadherent cells registered the least autoaggregation 11–20%. On the other hand, moderate adherent strains had a slightly high coaggregation index range of 44–77% followed by strong adherent 41–77% and nonadherent 12–40% strains (Table 2). It was also observed that 95% of the moderately adherent strains had a coaggregation index of >50% while the weak and strong adherent strains had 90% each (Table 2).

The partner strains *S. aureus*, *S. pyogenes *A, *P. shigelloides*, and *S. sonnei* displayed coaggregation indices of 75% each while *S. *Typhimurium, *A. hydrophila*, and *P. aeruginosa *registered coaggregation indices of 67%, 58%, and 50%, respectively. Isolate Liv 38-1 had the highest coaggregation range of 65–77% while the least was Liv 194-2, 12–28% (Table 3).

**Figure 1:**
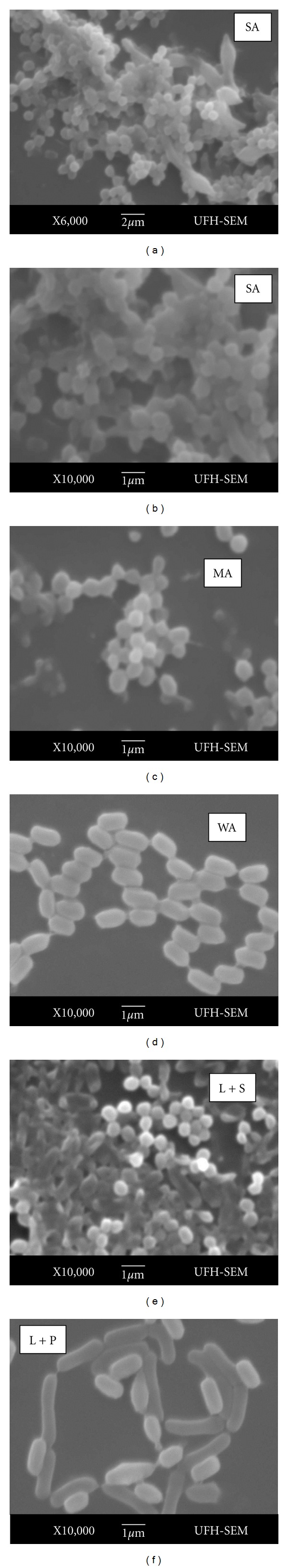
Scanning electron micrographs of autoaggregates and coaggregates biofilms of *L. ivanovii* and their coaggregates partner *S. aureus* NCTC 6571 and *P. aeruginosa* ATCC 15442. SA, strong adherent autoaggregate; MA, moderate adherent autoaggregate; WA, weak adherent autoaggregate; L + S, coaggregates of *L. ivanovii* and *S. aureus*; L + P, coaggregates of weak adherent *L. ivanovii *and *P. aeruginosa*.

In order to evaluate the architecture of the biofilms, SEM was used.  Figure 1 shows the scanning electron micrographs of autoaggregates and coaggregates biofilms of *L. ivanovii* and their coaggregates partner *S. aureus* NCTC 6571 and *P. aeruginosa* ATCC 15442. The different biofilm phenotypes were clearly distinguishable; the strong and moderate adherent strains (SA and MA) were seen as densely packed colonies while for the weak adherent strains (WA) few cells were stuck together and the cell morphology was clear (short thick rods), Figure 1. However, contrary to the microtiter results, it was observed that *L. ivanovii *isolates preferred to grow in single species than multispecies biofilm. 

## 4. Discussion

Control of foodborne pathogens to ensure food safety requires the consideration of many aspects of its natural and industrial ecology. Some *Listeria* spp. strains have been reported to be persistently present in environments for a range from eight months to ten years [19]. These resident strains are alleged to form biofilms in food processing equipment; the formed biofilms survive most processes used to kill microorganisms in food production; hence, increasing the chances of food contamination [20]. The present study was carried out to investigate the ability of *L. ivanovii* isolates to exist as single and mixed species biofilm structures. A number of methods have been developed for cultivation and quantification of biofilms; nevertheless, the microtiter plate method remains among the most frequently used assays for investigation of biofilm formation and quantification of bacterial biofilms. The study therefore used the microtiter plate assay to assess the ability of *L. ivanovii* strains to form biofilm. 

The potential of bacteria to form biofilms is affected by a number of factors including strain characteristics, physical and chemical properties of the solid phase, temperature, composition of growth medium, and the presence of other microorganisms [21]. Previous works have observed low biofilm quantities with tryptic soy broth [22, 23]; therefore this study used BHI broth which has been shown to strongly influence biofilm development in many organisms such as *Staphylococcus* and* Listeria* species [23, 24]. The present study observed that 88% of the strains were able to form biofilm at 25°C and four biofilm phenotypes were demonstrated. This is of great concern to the food industry especially in the tropics whose room temperature usually falls between 22 and 28°C; implying that with favorable conditions, these organisms at room temperature may grow and adhere to kitchen utensils or the environment if not properly cleaned, hence creating a source for cross-contamination. The attached cells in part also form a substrate for other microorganisms less prone to biofilm formation; this will lead to an increased survival rate of pathogen and further spreading during food processing. The findings concur those of Di Bonaventura et al. [25] who reported biofilm formation of *L. monocytogenes* at low temperatures (4, 12, and 22°C) on a glass. However, hydrophobicity was found to be higher at 37°C than at 4, 12, and 22°C.

Autoaggregation and coaggregation are of great importance in biofilm formation; they integrate biological structures, by mediating the juxtapositioning of species next to favorable partner species within taxonomically diverse biofilms. Autoaggregation is a process whereby a strain within the biofilm will utter polymers to boost the integration of genetically identical strains; these interactions are enhanced by increased hydrophobicity [17]. In the present study, isolates displayed variations in their autoaggregating abilities suggesting differences in strains and serotypes. 

Rickard et al. [26] defined coaggregation as a process by which genetically different bacteria become attached to one another via specific molecules. It was observed that *S. aureus*, *S. pyogenes *A, *P. shigelloides*, and *S. sonnei* were the strong partners while *P. aeruginosa*, a strong biofilm producer, recorded the least potential to coaggregate. The findings are in agreement with those of Jacobs and Chenia [27].

However, coaggregation results were contrary to SEM images where strong biofilms were observed in single species than in multispecies biofilms. Worthy of note is the fact that in autoaggregation assays, the individual isolates were grown separately, mixed, and incubated for only 60 minutes before the OD was read; while with the SEM the partner isolate was added after 2 hours of initial growth and the mixture was incubated for 72 hours. Previous studies have demonstrated the ability of *Listeria* species to grow on surfaces with other microorganisms, both Gram-positive and Gram-negative species, in a mixed species biofilm in food processing environments [24, 28]. However, Van der Veen and Abee [24] using plate counts and fluorescence microscopy showed that the cell count of *L. monocytogenes* was more than the partner strain, *Lactobacillus plantarum* cells. These findings are in agreement with our findings where few cells of the partner organism were apparent in a strong biofilm structures under SEM. Studies on *Flavobacterium* spp. observed that isolates which were unable to autoaggregate or showed low aggregation indices displayed varying levels of coaggregation with diverse aquatic bacteria [17]. In this study, the nonadherent strain (Liv 188-2) displayed both autoaggregation and coaggregation characteristics entailing that some of the strains though cannot attach to a solid surface as primary colonizers may interact with already formed organisms later as biofilm partners. Microorganisms can adhere to a surface where it acts as primary colonizers or as later biofilm partners by establishing interactions with other microorganisms [29]. Cell surface components (flagella, pili, adhesin proteins, capsules, and surface charge) are the major contributors to attachment and coaggregation in biofilms [27]. 

As crystal violet basically stains the number of cells that have attached to the wells of the microtiter, SEM analysis was employed to evaluate the architecture of the biofilms. Unlike weak biofilms where the morphology of single colonies were distinguishable, moderate and strong biofilms showed the presence of densely packed colonies of pleomorphic organisms (very short rods and coccobacilli); this could be in part that the cells were smaller due to competition hence they had to adjust for survival. This could explain the high level of resistance observed in biofilms as nutrient and oxygen depletion within the biofilm cause some bacteria to enter a stationary state, in which they are less susceptible to growth-dependent antimicrobial killing. Also some bacteria might differentiate into a phenotypically resistant state and express biofilm-specific antimicrobial resistance genes that are not required for biofilm formation but contributes to the survival of organisms in the biofilm.

## 5. Conclusion

The study demonstrated the ability of *L. ivanovii* isolates to form single and multispecies biofilms at 25°C with strong biofilms from single species. This is of great concern to the food industry where these organisms may adhere to kitchen utensils and the environment leading to cross-contamination. Some strains could not adhere to a surface but could autoaggregate and coaggregate implying that preventing primary adhesion would prevent biofilm formation in these strains. Future studies are required to determine the antimicrobial susceptibility of the biofilms as well as determine the virulence genes expression of adherence traits in these biofilms, to throw more light on their pathogenic potential in our environment.

## Figures and Tables

**Table 1:** Biofilm formation by *Listeria ivanovii* isolates (*n* = 50) following incubation at 25°C.

Biofilm formation	Number (%)	OD Range	Mean OD ± SD
Nonadherent	6 (12)	0.332–0.503	0.431 ± 0.055
Weak adherent	22 (44)	0.545–1.083	0.785 ± 0.175
Moderate adherent	17 (34)	1.105–2.084	1.432 ± 0.354
Strong adherent	5 (10)	2.32–3.846	3.045 ± 0.887
Total biofilm	44 (88)	0.545–3.846	1.754 ± 0.763

OD: optical density; SD: standard deviation. The results are the mean of three independent experiments carried out in triplicates.

**Table 2:** *Listeria ivanovii* isolates with coaggregation indices >50% among the four biofilm phenotype.

Biofilm phenotype	% Autoaggregation range	% Coaggregation range	Coaggregation indices > 50%
Non adherent	11–20	12–40	0
Weak adherent	30–46	37–75	90
Moderate adherent	35–41	44–77	95
Strong adherent	32–55	41–77	90

**Table 3:** Coaggregation indices of selected biofilm forming *Listeria ivanovii* isolates with seven different reference strains partners.

Isolate (biofilm phenotype)	% Autoagg.	Coaggregation indices (%)							
		Partner strains							
		Range %	*S. aureus *	*S. pyogenes* A	*S.* Typhimurium	*P. aeruginosa *	*P. shigelloides *	*A. hydrophila *	*S. sonnei *
			NCTC 6571	ATCC 49399	ATCC 13311	ATCC 15442	ATCC 51903	ATCC 35654	ATCC 29930
Liv 188-2 (NA)	19	18–37	31	28	18	37	24	27	19
Liv 119-2 (NA)	20	13–40	25	22	13	40	16	25	28
Liv 194-2 (NA)	11	12–28	19	12	17	28	13	20	16
Liv 37-1 (WA)	42	53–74	67	68	74	67	53	67	70
Liv 18-1 (WA)	30	37–70	54	51	45	37	70	40	66
Liv 01-2 (WA)	46	67–75	68	70	72	71	72	67	75
Liv 03-2 (MA)	41	61–71	74	67	71	61	64	68	71
Liv 155-1 (MA)	35	57–70	57	62	60	58	58	66	70
Liv 99-2 (MA)	36	44–77	51	55	52	53	77	44	60
Liv 16-2 (SA)	47	41–67	67	66	61	41	53	62	56
Liv 38-1 (SA)	32	65–77	72	73	77	69	71	65	72
Liv 41-1 (SA)	55	49–69	61	59	67	49	66	57	69

C. indices > 50%		50–75	75	75	67	50	75	58	75

NA: non adherent; WA: weak adherent; MA: moderate adherent; SA: strong adherent; Autoagg.: autoaggregation; C. indices: coaggregation indices > 50%.
